# Electromigration Techniques in the Analysis of Selected Cosmetic Ingredients: A Review

**DOI:** 10.3390/molecules30010161

**Published:** 2025-01-03

**Authors:** Joanna Kończyk, Magdalena Myga-Nowak, Rajmund Michalski, Katarzyna Janoszka

**Affiliations:** 1 Institute of Chemistry, Faculty of Science & Technology, Jan Dlugosz University in Czestochowa, Armii Krajowej 13/15, 42-200 Czestochowa, Poland; 2 Wladyslaw Bieganski Collegium Medicum, Jan Dlugosz University in Czestochowa, Armii Krajowej 13/15, 42-200 Czestochowa, Poland; m.myga-nowak@ujd.edu.pl; 3 Institute of Environmental Engineering, Polish Academy of Sciences, M. Skłodowskiej-Curie 34, 41-819 Zabrze, Poland; rajmund.michalski@ipispan.edu.pl (R.M.); katarzyna.janoszka@ipispan.edu.pl (K.J.)

**Keywords:** cosmetic, electromigration techniques, capillary electrophoresis, micellar electrokinetic chromatography, liquid chromatography

## Abstract

The cosmetics industry is one of the fastest-growing sectors worldwide. The dynamic evolution of this industry results in an increasingly diverse range of products containing various active ingredients. Ensuring the quality of these products is crucial for consumer safety, necessitating the use of advanced analytical methods and adherence to legal regulations. Electrophoretic techniques, particularly capillary electrophoresis and micellar electrokinetic chromatography, facilitate the rapid and precise separation and identification of cosmetic ingredients. A well-chosen technique and optimized analytical conditions ensure high sensitivity, repeatability, and resolution, achieving detection limits that meet legal requirements. Although electromigration techniques are less common in routine laboratory analyses compared to liquid chromatography, they show potential for broader application in analyzing various substances found in cosmetics. This study reviews the possibilities of applying different electrophoretic techniques to analyze selected cosmetic ingredients serving various functions, including preservatives, dyes, exfoliating agents, UV filters, and also contaminants, while considering sample preparation methods, equipment used, and analysis conditions. The compiled data indicate that capillary electrophoresis, when compared to high-performance liquid chromatography and ion chromatography, shows comparable or superior sensitivity and repeatability, with detection limits adequate to meet regulatory standards.

## 1. Introduction

The cosmetics industry is currently one of the most rapidly developing branches of industry in the world. The global cosmetics market value in 2021 amounted to EUR 425.5 billion, and a 5% average annual growth is forecasted for the years 2023–2026 [1]. In Poland, there are over 600 cosmetics manufacturers—large corporations with global brands as well as small producers—and the value of the Polish cosmetics market in 2023 amounted to PLN 25.4 billion, placing it fifth in Europe. In 2023, Poland was the ninth largest exporter of cosmetics in the world (with a 3.8% share of exports) and fifth in the European Union (8%). Cosmetics from Poland are exported to over 160 countries, both within the European Union (including Germany, France, Spain, and the United Kingdom) and to distant countries such as the United States, Canada, Mexico, Indonesia, and Australia [2]. This development translates into an increasingly wide range of cosmetics and cosmetic products on the market. According the Regulation (EC) No 1223/2009 of the European Parliament and of the Council of 30 November 2009, “a ‘cosmetic product’ means any substance or mixture intended to be placed in contact with the external parts of the human body (…), with a view exclusively or mainly to cleaning them, perfuming them, changing their appearance, protecting them, keeping them in good condition or correcting body odors”. Manufacturers offer products with more or less complicated compositions, containing well-known or completely new active substances, of natural or synthetic origin, dedicated to specific consumer needs. It is estimated that the average woman uses 12 cosmetic products daily, containing a total of up to 168 ingredients, while the average man uses six cosmetic products containing a total of approximately 85 ingredients [3].

Cosmetic formulations are created to meet consumer expectations and a range of rigorous requirements regarding safety, application properties, and effectiveness. A cosmetic contains base substances (e.g., water, alcohols), form-stabilizing agents (e.g., preservatives, antioxidants), active ingredients (e.g., surfactants, UV filters), or auxiliary substances (e.g., coloring agents, fragrances). Many of these substances have limitations regarding their percentage content in the final product. An example is preservatives (e.g., sodium benzoate, phenoxyethanol, potassium sorbate, benzyl alcohol, esters of 4-hydroxybenzoic acid), whose presence is essential in cosmetics containing water. Preservatives provide protection of the cosmetic product against the development of bacteria and fungi, thereby ensuring the safety of the product throughout its shelf life [4]. On the other hand, coloring substances are intended exclusively or mainly for coloring the cosmetic product (mainly for aesthetic purposes), the entire body (e.g., self-tanners), or certain parts of it (lipsticks, powders, pencils, mascaras). They are used in the form of dyes, which permanently bind to the colored surface, or coloring substances that are applied to the surface temporarily. On cosmetic product labels, in the list of ingredients, dyes and pigments, with the exception of hair dyes, are listed under the CI (Color Index) identification number of the given dye [5]. Substances absorbing UV light serve as UVA and UVB filters. Among them are physical filters, known as mineral filters, such as zinc oxide (ZnO), titanium dioxide (TiO_2_), and chemical filters, which include the following organic compounds: benzophenones, cinnamates, derivatives of p-aminobenzoic acid, and salicylates.

In order to confirm the presence of a given ingredient in a cosmetic product and its quantity, an appropriate method of analysis should be applied to ensure good quality control. Frequently, due to the high complexity of the matrix, it is necessary to properly prepare the tested cosmetic sample prior to the analysis. Many analytical techniques are used for the analysis of cosmetics, including chromatographic (high-performance liquid chromatography—HPLC [6,7], gas chromatography—GC [7,8], ion chromatography—IC [9,10]), electromigration (capillary electrophoresis—CE [11], micellar electrokinetic chromatography—MEKC [12]), spectrometric (infrared spectrometry—IR [13,14], atomic absorption spectrometry—AAS [15], coupled with inductively coupled plasma: optical emission spectrometry—ICP-OES or mass spectrometry—ICP-MS [16]), while the technique selection depends on the type and nature of the analyte and the sample [17,18].

Electromigration techniques offer significant advantages for analyzing ionic and polar compounds in complex sample matrices compared to other methods. Unlike chromatographic techniques such as HPLC, IC, or GC, electromigration methods require smaller amounts of solvents and reagents, making them more environmentally friendly and cost-effective. They also provide high separation efficiency and resolution with faster analysis times, which is particularly advantageous for high-throughput testing in quality control or regulatory compliance checks. In contrast to spectrometric methods, which often involve complex sample preparation, electromigration techniques can analyze intricate cosmetic matrices, such as creams or lotions, with minimal preparation. Additionally, their ability to work with very small sample volumes is beneficial when handling expensive or limited samples, such as luxury cosmetics or rare formulations. The combination of speed, efficiency, eco-friendliness, and minimal sample preparation makes electromigration techniques highly suitable for modern cosmetic analysis.

The aim of this study was to review the literature on electromigration techniques and the possibility of their application for the qualitative and quantitative determination of cosmetic ingredients.

## 2. Legal Standards for Cosmetic Quality

The quality of a cosmetic product entering the market is a key factor determining the safety of its users; therefore, it must meet the requirements specified in relevant legal acts. In the European Union countries, the requirements for cosmetic products have been specified in Regulation (EC) No 1223/2009 of the European Parliament and of the Council of 30 November 2009, with subsequent amendments. This document standardized the regulations and guidelines regarding control, safety, liability, and documentation related to the production and distribution of cosmetics, detailing prohibited substances in cosmetics (Annex II covering 1730 entries), substances that may be included in cosmetics in limited amounts (Annex III covering 378 entries), permitted colorants (Annex IV covering 153 entries), preservatives (Annex V covering 60 entries), and UV filters (Annex VI covering 34 entries). This regulation is also in force in Poland, and it is complemented by a number of national regulations concerning detailed guidelines for the trade of cosmetic products (Journal of Laws 2018 item 2227, Journal of Laws 2019 item 350, Journal of Laws 2019 item 417). Furthermore, all UV filters allowed for use in cosmetic products have been positively evaluated in terms of safety by the Scientific Committee on Consumer Safety (SCCS) and the U.S. Food and Drug Administration (FDA) [19]. The Regulation of the Minister of Health of 19 March 2020 (Journal of Laws 2020 item 931) presents detailed requirements for the analysis of selected cosmetic ingredients, taking into account the sample preparation stage, analytical techniques, and methods of analysis.

EU regulations impose on manufacturers and importers the obligation to conduct detailed testing of the raw materials used and the cosmetic products offered. While large corporations have their own analytical laboratories, small enterprises and individual consumers use external laboratory services. Year by year, the number of commercial research laboratories offering detailed quality assessments of cosmetic raw materials and cosmetics is increasing, based on determining their basic quality parameters, microbiological purity, or chemical composition. The tests performed in these laboratories are based on methodologies described in relevant standards indicated by the Polish Committee for Standardization, regulations, or their own methods—developed based on scientific research or application notes from equipment manufacturers, e.g., [20].

## 3. Electromigration Techniques

### 3.1. Division of Electromigration Techniques

**Figure 1:**
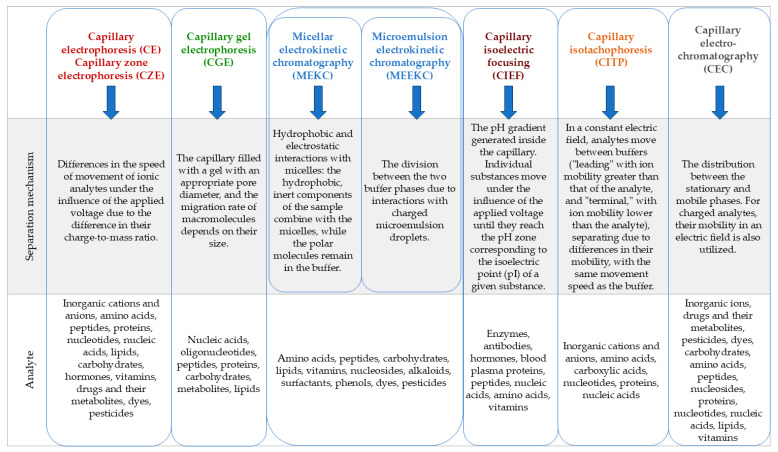
Division and characterization of electromigration techniques (based on [21,30,31,32]).

Electromigration techniques are techniques for qualitative and quantitative analysis of multicomponent samples, based on the phenomenon of particle migration through a capillary filled with a conductive liquid medium called a buffer or electrolyte (often referred to as BGE—*Background Electrolyte*), in an electric field [21]. Due to the method of conducting the separation process, several types of electromigration techniques can be distinguished, according to the scheme presented in Figure 1. Since the introduction of modern capillary electrophoresis (called high-performance capillary electrophoresis) by Jorgernson and Lukacs in 1981 [22], this technique has gradually evolved to eventually become a comprehensive analytical technique of interest to scientists, which is confirmed by numerous scientific works on both theoretical issues, new technical solutions, including miniaturization [23,24], and its applications in various areas of life, such as medicine, pharmacy, cosmetology, the food industry, and environmental protection [25,26,27,28,29].

### 3.2. Electromigration Techniques and Liquid Chromatography—A Comparison

Due to the complex chemical composition of cosmetics, which often complicates the correct identification and quantitative analysis of specific ingredients or potential contaminants, as well as legal requirements, it is important that the analytical method used meets certain criteria. In this context, it is essential to compare electromigration techniques with more commonly used chromatographic methods. Both types of methods have unique features and capabilities that determine their application in cosmetic analysis; however, they also have certain limitations that should be considered when choosing the appropriate method.

Capillary electrophoresis (CE) and ion chromatography (IC) are two powerful analytical techniques used for the separation and detection of ionic components. Due to their similar application areas, these methods were the subject of comparative studies in ion determination [33,34,35]. Currently, ion chromatography is recognized as a reference method for determining basic inorganic cations and anions in drinking water and wastewater [36,37]. This has led to its more frequent use in determining ionic components in other matrices, including studies on the quality of cosmetic raw materials and the ionic composition of cosmetics [38,39,40]. Although CE and IC have some similarities, they differ significantly in terms of fundamental operating principles and performance characteristics, which makes the scope of their application possibilities different.

Both CE and IC aim to separate ions in a mixture, but this separation results from different mechanisms. CE separates ions based on their differential migration time in an electric field, while IC relies on differences in ion affinity to the stationary ion-exchange phase, leading to different profiles of analysis efficiency. CE is characterized by excellent separation efficiency of analytes and shorter analysis times, while consuming a small amount of the sample and reagents, making it a more environmentally friendly technique and, in terms of laboratory and industrial research, more economical. On the other hand, IC, although slower and requiring larger sample volumes, exhibits higher sensitivity, especially for anion analysis, and can be easily used for analyzing high-conductivity samples. CE is more versatile and generally cheaper, but its sensitivity, especially for anions, is lower, and its application is limited to low-conductivity samples. Additionally, temperature fluctuations significantly affect CE, whereas IC remains relatively unaffected by these changes.

Similar to IC, high-performance liquid chromatography (HPLC) also exhibits versatility and precision in analyzing components of varying polarity and molecular weight. The versatility of HPLC lies in its ability to identify and quantitatively analyze a wide range of compounds, from small organic molecules to large polymer compounds, making it invaluable in the analysis of cosmetics with complex matrices [41,42,43,44,45]. Although slower than CE, the HPLC technique is highly sensitive, and the use of various detectors enhances its analytical capabilities.

Electromigration techniques, that are inherently more environmentally friendly and economical due to low solvent and reagent consumption, are ideal for rapid separation analyses and preliminary sample cleaning. On the other hand, analyses requiring high precision and the ability to separate more complex mixtures and non-volatile components are better conducted using chromatographic techniques. Furthermore, chromatography is often more useful in routine analyses due to better-defined standards, protocols, and greater equipment availability in industrial laboratories. When comparing both groups of techniques, it should be emphasized that the choice between them should be closely related to the specific requirements of the analysis (type of analyte, type of matrix, required sensitivity, complexity of sample preparation, costs, analysis time, and result processing) [46]. Validation parameters obtained during HPLC and CE analyses of hexachlorophene content in cosmetics conducted by Li et al. [41] indicate a slight advantage of the HPLC method over CE (quantification limits (LOQ) are 0.19 µg/mL and 0.15 µg/mL, average recoveries—92.3% and 102.2%, and average relative standard deviations (RSD)—1.7% and 0.32%, respectively, for CE and HPLC), and the nearly three times shorter analysis time of CE (5 and 14 min, respectively, for CE and HPLC) suggests choosing this technique as more economically advantageous. Although the HPLC analysis of parabens in cosmetics [42] required almost twice the analysis time, in this case, the chromatographic method was characterized by, depending on the analyte, a four to eight times lower detection limit than CE. In turn, the advantage of ITP compared to HPLC is the lack of necessity for preliminary sample preparation and derivatization, which makes the analysis costs using this method lower. However, a disadvantage of ITP is the relatively high detection limit values [47].

The limitations of electromigration techniques can be mitigated by using chromatographic techniques or vice versa. Such synergy used in complex analytical studies allows for obtaining the full chemical composition of the tested cosmetic, including information about its purity and safety, and indirectly also on the correctness of the technological process conducted.

## 4. Application of Electromigration Techniques in Cosmetic Analysis

**Figure 2:**
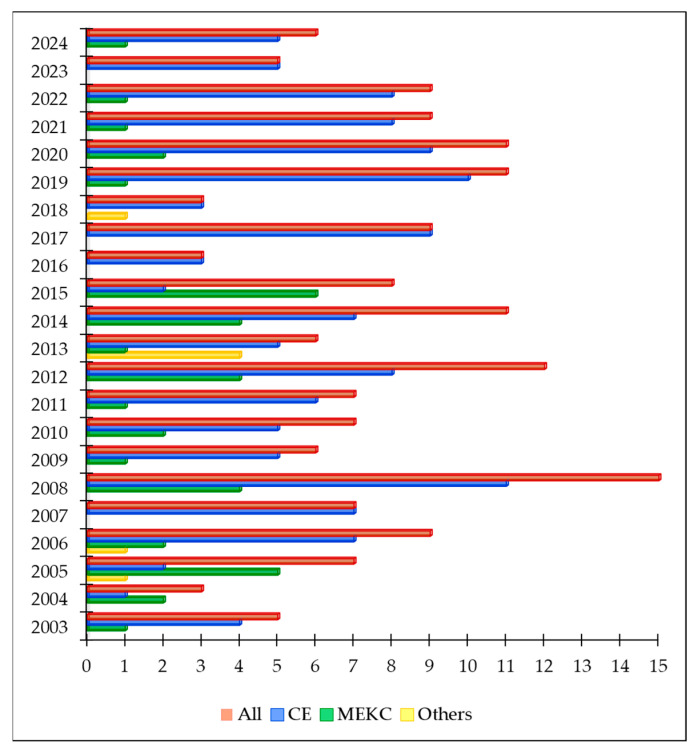
The number of publications from 2003, according to the Scopus database (keywords: for CE—cosmetics AND “capillary electrophoresis”; for MEKC—cosmetics AND “micellar electrokinetic chromatography”).

Every year, new studies on the analysis of the composition of cosmetics using electromigration techniques, mainly capillary zone electrophoresis (marked in the literature with the abbreviation CE or CZE) and micellar electrokinetic chromatography (MEKC) appear in the scientific literature databases (Figure 2). Although this is not as impressive a figure as that of liquid chromatography (with over 2000 articles in the past 10 years), the existence of these works highlights the need to employ electrophoretic techniques in areas where HPLC chromatography encounters certain limitations.

### 4.1. Preservatives

The most commonly used preservatives in cosmetics are parabens, which are alkyl esters of 4-hydrocybenzoic acid, especially methylparaben (MP), ethylparaben (EP), propylparaben (PP), butylparaben (BP), isobutylparaben (IBP) or benzylparaben (BZP). These are substances with a broad antimicrobial effect, good stability over a wide pH range, and low volatility. Due to synergistic effects, cosmetics often contain two or more parabens. Recent studies indicate the harmful effect of parabens on the human body (e.g., allergies, disorders of the endocrine system); therefore, the applicable law allows for the use of 4-hydroxybenzoic acid, its salts and esters, in limited amounts—not exceeding 0.4 wt% for a single paraben and 0.8 wt%—for a mixture of parabens, expressed as acid concentration.

**Figure 3:**
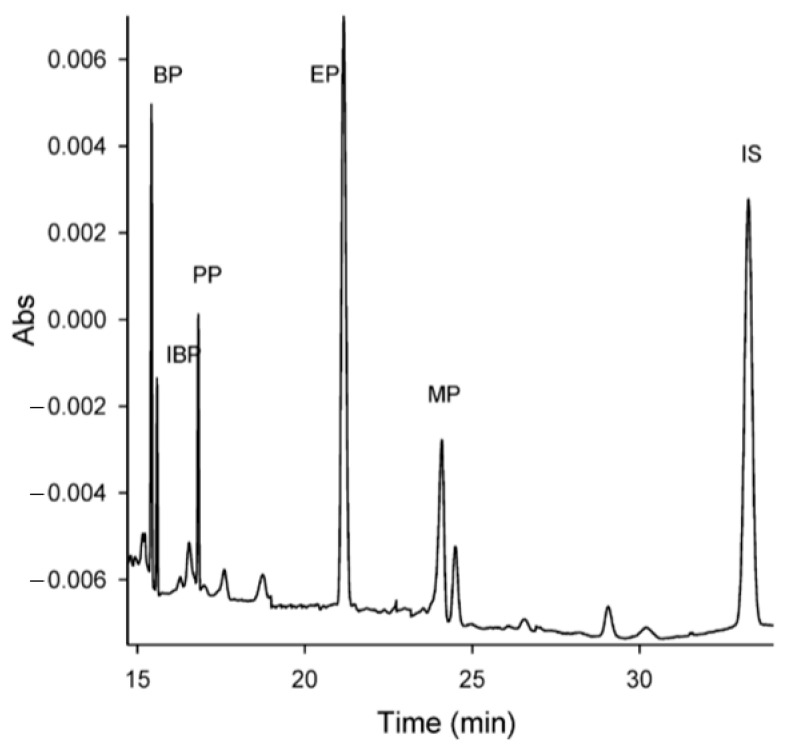
Electropherogram of parabens (BP—butyl, IBP—isobutyl, PP—propyl, EP—ethyl, MP—methyl) in commercial cosmetic product (IS—internal standard). Reprinted from [50], with permission from Wiley.

There is a lot of research in the literature on the possibility of using electromigration techniques for the identification and concentration analysis of parabens in cosmetic products [48,49,50,51,52,53,54,55,56]. Some of these studies, along with the methodologies used, are listed in Table 1, and example electropherograms are shown in Figure 3.

The most common method for determining parabens is capillary electrophoresis with spectrophotometric detection in a variable wavelength detector (UV) [48,49,50] or a diode array detector (DAD) [51,52] at wavelengths ranging from 200 to 298 nm, at a temperature of 20 or 25 °C, in quartz capillaries with an internal diameter of 50 µm. Optimization of the composition of the buffers BGE used for the analyses allowed for the qualitative and quantitative analysis of both parabens and several other preservatives, such as: sorbic acid (SOA), benzoic acid (BA), salicylic acid (SA), p-hydroxybenzoic acid (HBZA), and dehydroacetic acid (DHOA).

The micellar electrokinetic chromatography technique MEKC with borate buffer (BB) [53,54,55] or phosphoric acid buffer [12] modified with sodium dodecyl sulphate (SDS), and capillary electrochromatography (CEC/DAD), with a capillary filled with 5 µm Lichrospher 100 C18 phase [56] has also been successfully used for the analysis of parabens.

Cosmetics in the form of cream, toner, lotion, gel, shampoo, powder, or paste are samples with complex matrices that require appropriate preparation for analysis. Cabaleiro et al., in their publication [57], reviewed the methods of preparing samples containing parabens for their analysis using various techniques, including electromigration. In turn, Maijo et al. [58] determined the effect of the type of analyte concentration technique used on the values of the validation parameters of the method used (CE or MEKC), using the following strategies: large volume sample stacking (LVSS), field amplified stacking injection (FASI), and sweeping and in-line solid-phase extraction–capillary electrophoresis (in-line SPE–CE). In the case of electrophoretic concentration, the obtained limit of detection (LOD) values ranged from 18 to 27; from 3 to 4 and were equal to 2 ng/mL, respectively, for sweeping, LVSS and FASI, while for the in-line SPE–CE method, these limits were significantly lower (from 0.01 to 0.02 ng/mL). Classic ultrasonic extraction of parabens from shampoo foam using an ether–acetic acid mixture [42] allowed for a quantitative, quick (10 min) and accurate analysis of MP, EP and PP parabens; however, under the applied analysis conditions, it was impossible to separate the isomeric forms of butyl paraben (BP and IBP). The analysis of the same samples by means of HPLC allowed for the separation of these isomers, and the analysis time was two times longer. The obtained LOD values for the HPLC technique were significantly lower (from 20 to 50 ng/mL) than those obtained for the CE (160–210 ng/mL), but nevertheless higher than those obtained with the FASI–CE technique [58]. Ye et al. [48] proposed the sorption of parabens using graphene as a SPE sorbent, and then their desorption from the sorbent with methanol. This method of sample preparation obtained LOD values in the range from 100 to 150 ng/mL, which was slightly lower compared to the ultrasonic extraction proposed by Labat et al. [42]. The CE technique with simultaneous stacking with a large sample zone and sweeping made it possible to determine the parabens extracted from the cream with methanol [50]. Using BGE buffer containing 50 mM phosphate buffer (PB) with pH 3.0; 80 mM SDS and 30% methanol, it was also possible to separate butyl paraben from isobutyl paraben.

The method used by Xue et al. [49] based on dispersive liquid-liquid micro extraction coupled with capillary electrophoresis (DLLME–CE) turned out to be a fast, cheap and effective method of analyzing both hydrophobic preservatives, such as parabens: MP, EP, BP and PP, and hydrophilic preservatives, such as sorbic (SOA), benzoic (BA) and salicylic (SA) acids, in creams and lotions. Compared to high-performance liquid chromatography combined with microwave assisted extraction (MAE–HPLC), DLLME–CE obtained 10 times lower LOD values (0.2 mg/kg for EP and BA and 0.375 mg/kg for other analytes, with two times shorter extraction time (18 min).

The use of the LVSS technique in MEKC made it possible to achieve a much higher sensitivity and resolution of paraben analysis compared to the classic MEKC analysis [12]. On the other hand, MEKC combined with the sweeping technique, under optimal analysis conditions, turned out to be useful for determining the concentration of parabens next to the whitening ingredients of cosmetics (hydroquinone, arbutin, kojic acid, resorcinol, and salicylic acid), with detection limits lower than the maximum concentration level for the tested compounds determined by applicable legal acts [54]. Qualitative and quantitative analysis of parabens in cosmetics is also possible using microemulsion electrokinetic chromatography [55] and capillary electrochromatography [56]. Nevertheless, to achieve high sensitivity, repeatability, and resolution of the analysis, the conditions for its application should be carefully selected.

### 4.2. Exfoliating and Moisturizing Substances

One of the groups of chemical compounds appearing in a cosmetic as an exfoliating agent is α-hydroxy acids, also called fruit acids (AHA), i.e., organic acids with hydroxyl groups attached to the carbon atom adjacent to the carboxyl group, found naturally in most fruits, milk, sugar cane juice, wine, and beer. For the production of cosmetics, lactic, malic, glycolic, citric, almond, or tartaric acids are most frequently used [59,60]. In cosmetic products, these acids are usually present in concentrations from 4 to 10%, with the exception of chemical peeling, in which they are present in concentrations above 20%. The recommended daily AHA use limit that does not threaten the consumer is 10%; therefore, it is necessary to control the content of these compounds in cosmetic products.

Among electromigration techniques, capillary electrophoresis with UV [11,61] or DAD detection is most frequently used for qualitative and quantitative confirmation of the presence of these compounds in cosmetic products (Table 2).

In order to achieve a better separation of AHA acids, Liu et al. [62] and Chen et al. [63] applied an additive to the buffer in the form of γ-cyclodextrin (γ-CD), which changes the mobility of the analytes due to the formation of AHA-CD complexes.

Among the numerous ingredients of moisturizing cosmetics, two groups can be distinguished: (1) hydrophilic compounds, e.g., collagen, hyaluronic acid, sorbitol, urea, and (2) hydrophobic compounds, which include, among others, fatty acids and ceramides. Literature reports indicate the possibility of using electromigration techniques to analyze the content of selected compounds from both groups. Selected examples of such analyses are presented in Table 2.

Hyaluronic acid, which is a linear polysaccharide composed of alternately repeating units of D-glucuronic acid and *N*-acetyl-D-glucosamine linked by β-1,4 and β-1,3-glycosidic bonds, respectively, was the subject of research by Lin et al. [64] on the selection of MEKC analysis conditions, enabling the simultaneous analysis of low-molecular and high-molecular hyaluronic acid in a cosmetic matrix using Olivem 1000. Under the optimal analysis conditions presented in Table 2, it was possible to separate the peaks of the tested analytes within 10 min, with 98.2% and 95.3% analyte recovery, and detection limits of 2 and 10 µg/mL, for low- and high-molecular acid, respectively. In turn, Chindaphan et al. [65] proposed CE with a large volume sample stacking using an electro-osmotic flow pump (LVSEP), with a detection limit of 3 µg/mL, to determine the content of this acid in cosmetics.

**Figure 4:**
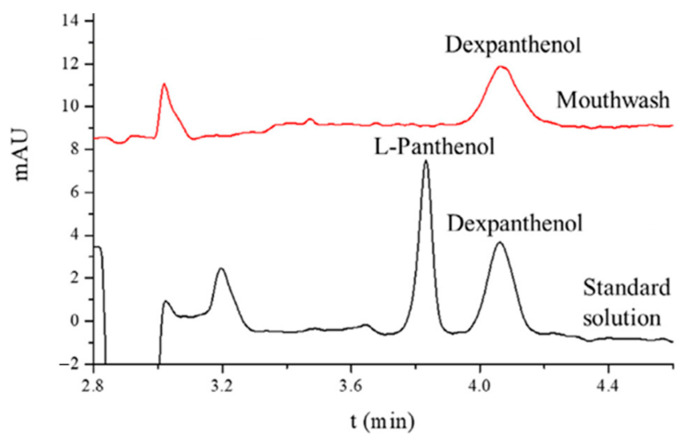
Electropherograms of L-panthenol and dexpanthenol in their standard solution and mouthwash. Reprinted from [68], with permission from Elsevier.

Examples of the use of electromigration techniques for the determination of moisturizing substances of a hydrophobic nature are the tests described in the publications [66,67] presenting the possibilities of capillary electrophoresis for the analysis of fatty acids: palmitic, stearic, oleic, and linoleic. Considering the fact that the fatty acids used in cosmetics come mainly from vegetable oils, the analysis of their composition should be the first step in the preliminary assessment of the quality of such raw material and its suitability for the production of a cosmetic. The methodology for detecting and separating enantiomers of panthenol (L-panthenol and dexpanthenol) using cyclodextrin electrokinetic chromatography (CD-EKC) was developed by Jiménez et al. [68]. Under optimal analytical conditions—25 mM (2-carboxyethyl)-β-CD (CE-β-CD) in 100 mM borate buffer (pH 9.0), a voltage of 30 kV, and a temperature of 30 °C—the analytes were separated within 4.2 min, achieving a resolution of 2.0, with LOQs of 1 mg/L and 4 mg/L for the L- and Dex-enantiomers, respectively (Figure 4). This demonstrates that the developed method meets the requirements of the International Council for Harmonization of Technical Requirements for Pharmaceuticals for Human Use (ICH) for detecting contaminating L-panthenol (0.1% relative to the predominant enantiomer).

### 4.3. Substances Absorbing UV Light

Sunscreen cosmetics contain substances that act as filters of ultraviolet radiation, which is harmful to the skin. Benzophenones, and phenylbenzotriazoles, as well as p-aminobenzoic, p-methoxycinnamic, salicylic acids and their derivatives are used as chemical UV absorbents [69]. Examples of the methodologies for analyzing this group of cosmetic ingredients are presented in Table 3.

**Figure 5:**
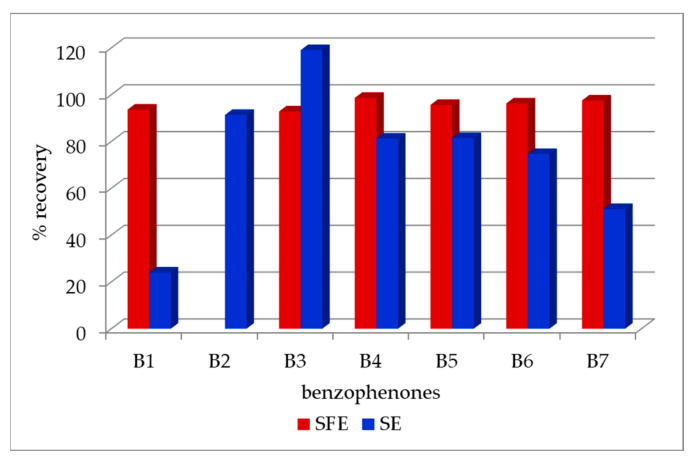
Comparison of the efficiency of benzophenone extraction from cosmetics using supercritical fluid extraction (SFE) and solvent extraction (SE) techniques (based on data from [70]).

Electromigration techniques such as CE, MEKC and MEEKC have been successfully applied to the analysis of benzophenones [43,70,71], p-aminobenzoic acid, and 2-hydroxy-4-methoxybenophenone-5-sulfonic acid [43]. Wang et al. [70] analyzed seven benzophenones in commercially available sunscreen cosmetics using CE with sample pretreatment by supercritical fluid extraction (SFE) using CO_2_ with 2.5% modifier in the form of a mixture of 10% phosphoric acid and methanol (1:1). Although SFE failed to extract 2,2′,4,4′-tetrahydroxybenzophenone (B2), this technique allowed for the extraction of larger amounts of five out from remaining six benzophenones from cosmetics, compared to the classic SE solvent extraction (Figure 5), and their CE analysis took less time than HPLC analysis. It was also found that adding the non-ionic surfactant Tween 20 to the buffer increased the peak resolution of the analyzed benzophenones.

A three times shorter analysis time for CE compared to HPLC was achieved by Juang et al. [43] for eight UV absorbers, with simultaneously good reproducibility of the proposed method, expressed through RSD in the range of 1.65–3.55%, and LODs of individual analytes from 0.23 to 1.86 µg/mL. Optimization of conditions for MEKC and MEEKC also allowed for the separation and detection of benzophenones in cosmetic samples [71]. It was found that the concentration of SDS added to the buffer and the column temperature do not significantly affect the resolution of benzophenones separation in the case of MEEKC, but they are important in the case of MEKC. In addition, the buffer pH and the presence of ethanol as an organic modifier significantly affect the separation selectivity for both techniques, and increasing the applied electric voltage improves the separation efficiency, while reducing the separation resolution for MEKC, without a noticeable reduction in separation resolution for MEEKC.

The use in MEEKC of a buffer containing two surfactants, anionic SDS and neutral Brij 35 and an organic modifier in the form of 2-propanol, also allowed for qualitative and quantitative analysis (with LOD ranging from 0.8 to 6.0 µg/mL) of other cosmetic ingredients absorbing UV light, included in the group of absorbers under the trade name Eusolex: Eusolex 4360 (ethylhexyl methoxycinnamate), Eusolex 6300 (butyl methoxydibenzoylmethane, Eusolex OCR (octocrylene); Eusolex 2292 (ethylhexyl salicylate); Eusolex 6007 (diethylamino hydroxybenzoyl hexyl benzoate, Eusolex 9020 (ethylhexyl triazone), Eusolex HMS (bis-ethylhexyloxyphenol methoxyphenyl triazine), Eusolex OS (ethylhexyl methoxycinnamate), and Eusolex 232 (octyl salicylate) [72].

### 4.4. Antioxidants

Antioxidants, often regarded as a “miracle” ingredient in cosmetics due to their ability to rejuvenate cells and prevent skin cancer, can pose potential health risks when used in excess. For this reason, alongside the need for tests to exclude prohibited antioxidants, the quantity of permitted ones should also be monitored. Table 4 presents selected examples of antioxidant analyses in cosmetics using electrophoretic techniques.

Many synthetic antioxidants, such as propyl gallate (PG), *t*-butyl hydroquinone (TBHQ), butylated hydroxyanisole (BHA), and butylated hydroxytoluene (BHT) are used in the manufacture of skin care products. To determine PG and TBHQ in cosmetic samples, the CE technique with amperometric detection (CE-EC) was proposed, using a porous connection that eliminates the influence of high-voltage electrophoretic field on the EC detector with a three-electrode system (carbon fiber electrode/platinum electrode/silver chloride electrode) and the analysis conditions listed in Table 4 [73]. An electrochemical detector has also been successfully used for the simultaneous analysis of all four of the above-mentioned antioxidants, carried out using the MEKC technique [74]. To achieve effective quantitative determination of carnosine and niacinamide in cosmetic formulations (a whitening essence sample and an antiglycation pill), Chen et al. [75] utilized CE with microchips made from cyclic olefin copolymer (COC) featuring dynamic and static coatings. The static coating was developed through the adsorption, immobilization, and closure of active sites of bovine serum albumin, which enhanced the hydrophilicity of the COC surface and minimized nonspecific peptide adsorption. Meanwhile, the dynamic coating was formed by adding a surfactant to the buffer, playing a key role in regulating flow rate and improving column efficiency in the separation channel. The analyses were performed using a 0.1 mM sodium tetraborate buffer with 0.03% SDS, achieving analyte separation within 30 min, with recoveries ranging from 80.9% to 112.6% and detection limits of 0.09 mg/kg for carnosine and 0.17 mg/kg for niacinamide. In turn, L-ascorbic acid and its derivatives, magnesium salt of ascorbic acid-2-phosphate and ascorbic acid-6 palmitate, were simultaneously determined using MEKC [76]. Optimization of the analysis parameters and buffer composition allowed for the separation and quantitative analysis of these whitening agents in cosmetics in less than 15 min, achieving good precision and accuracy of the applied method (RSD < 3%).

### 4.5. Dyes

**Figure 6:**
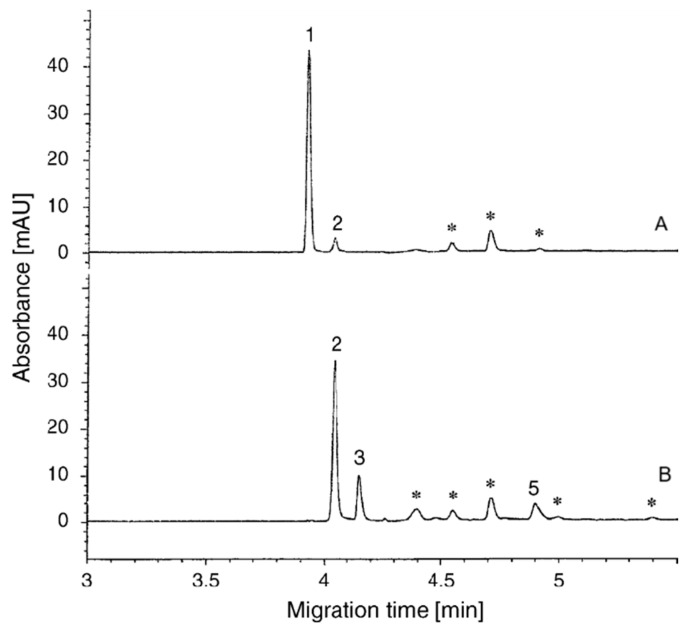
Electropherograms of basic dyes (1—Basic Red 76; 2—Basic Brown 16; 3—Basic Yellow 57, 5—Basic Blue 99) in hair coloring gels (A and B); ∗—other unidentified sample components. Reprinted from [77], with permission from Elsevier.

A large group of cosmetics available on the market is the so-called color cosmetics (nail polishes, powders, shadows, lipsticks, blushes, etc.) and cosmetics with coloring properties (hair dyes, mousses and gels for dyeing hair), whose color is due to the presence of numerous coloring substances in them in the amounts regulated by relevant legal acts. Details of exemplary analyses of this group of cosmetics ingredients are presented in Table 4. The CE technique with a buffer with the optimized composition: 100 mM acetate buffer + 90% vol. methanol (pH = 6.6) was used to determine five basic dyes from the group of the so-called Arianols present in hair dyes, and the results of this method’s validation (%RSD: 3.7–5.9%; LOQ: 2.3–14.8 µg/mL; % recovery: 93.3–111.2%) indicate a sensitivity, precision and accuracy sufficient to be used to control the quality of hair care products [77]. It was noted that washing the entrance end of the capillary with 90% (*v*/*v*) methanol immediately after injecting the sample prevented the peaks from tailing (Figure 6).

Gładysz et al. [78] proposed the MEKC analysis methodology with DAD detection, sample preparation by ultrasound-assisted extraction and a repeatable procedure of taking small amounts of the sample using a specially designed and constructed device for the identification of eight dyes present in red lipsticks from different manufacturers. In turn, Sun et al. [79] proposed a novel approach to open-tubular capillary electrophoresis (OT-CEC) using the diblock copolymer poly(butyl methacrylate)_71_-block-poly(glycidyl methacrylate)_9_ (P(BMA)_71_-b-P(GMA)_9_ for separating aromatic amines in nail polishes. Compared to traditional capillaries, this innovative coating material significantly enhanced separation performance, with detection limits of 13.6 µM for aniline, 7.2 µM for p-nitrylaniline, and 9.9 µM for α-naphthylamine.

### 4.6. Other Cosmetics Ingredients and Contaminants

In addition to the applications of electromigration techniques for analyzing cosmetic compositions mentioned in the previous sections, the literature also proposes the use of CE with different detectors, MEEKC and CITP, for examining specific cosmetic ingredients and impurities. The conditions for conducting such analyses are presented in Table 5. CE systems with UV detection with borate buffer (BB) were successfully utilized for determination of hexachlorofene [41], anionic, cationic, and amphoteric surfactants [44], as well as chelating agents, such as ethylenediaminetetraacetic acid, ethylenediamine-disuccinic acid, and iminodisuccinic acid [45], in different cosmetics. Meanwhile, the CE-DAD technique with MES/MOPSO/TTAB buffer proved most effective for analyzing silver nanoparticles in face cream [80].

Martínez-Girón et al. [81] analyzed perfumes for the presence of polycyclic musks, galaxolide, tonalide, traseolide, and phantolide, using a MEKC system with chiral cyclodextrin selectors (CD-MEKC) and a 2-[*N*-cyclohexylamino]ethane sulfonic acid (CHES) buffer supplemented with SDS. Through method optimization, separation of the analytes in under 10 min for tonalide, 13 min for traseolide and phantolide, and 17 min for galaxolide was achieved. Furthermore, a sweeping injection strategy was employed to enhance the method’s sensitivity, enabling a concentration factor increase of up to 12 times compared to the conventional injection method.

Capillary electrophoresis coupled with tandem mass spectrometry with inductively coupled plasma (ICP-MS/MS) allowed Zajda et al. [82] to investigate the encapsulation of a copper tripeptide complex (GHK–Cu) in liposomes. This analytical setup provided effective separation of all sample components, which were then detected using isotope-specific ICP-MS/MS, enabling simultaneous analysis of copper as a marker of the active cosmetic ingredient and phosphorus as a marker of the liposome. In addition to the intended ingredients of cosmetics, their composition may also include impurities, the source of which may be a cosmetic raw material with insufficient purity used in production, a production device from which they are released, or deliberately adding them to the cosmetic.

Ni et al. [83] developed a quick and simple method for the analysis of MEEKC of seven structurally similar corticosteroids (prednisone, hydrocortisone, prednisolone, hydrocortisone acetate, cortisone acetate, prednisolone acetate, and triamcinolone acetonide) in samples of selected cosmetics, using the ionic liquid 1-butyl-3-methylimidazolium tetrafluoroborate (BmimBF_4_). A synergistic effect was observed between the ionic liquid and the surfactant SDS, resulting in a reduction of the concentration of surfactants above which micelles are spontaneously formed. It was possible to separate the analyzed corticosteroids in approximately 28 min, with the following validation parameters: recoveries ranging from 86 to 114%, RSD below 4.8%, and LODs from 1.9 mg/L for prednisolone to 3.5 mg/L for hydrocortisone acetate.

The presence of such substances in a cosmetic as toxic heavy metals (e.g., Cd, Pb and Hg) or phenolic compounds (bisphenol A, α- and β-naphthol, 2,4-dichlorophenol) may have a very negative effect on the human body; therefore, the scope of cosmetic quality control should also include these substances. An example of the use of electromigration techniques for the analysis of heavy metals in cosmetics is the research of Chen et al. [84]. They developed a technique of sample preconcentration by sweeping through dynamic chelation before the actual Pb, Hg and Cd analysis with CE. The result of the application of such methodology was the quantitative analysis of lead, cadmium and mercury ions in cosmetics with the detection limits of 15, 50 and 100 ng/mL for Pb, Hg and Cd, respectively. In turn, Zhou et al. [85] proposed CE/UV with a two-stage sample preparation consisting of dispersive microextraction of analytes in a mixture of ionic liquid and acetone (IL-DLLME) and their re-extraction into sodium hydroxide solution for the determination of phenolic compounds in aqueous cosmetics. Under the optimal conditions of the experiment, the concentration coefficients and detection limits were, respectively, 60.1 and 5 ng/mL for bisphenol A, 52.7 and 5 ng/mL for β-naphthol, 49.2 and 8 ng/mL for α-naphthol, and 18.0 and 100 ng/mL for 2,4-dichlorophenol.

## 5. Summary

Although capillary electrophoresis is often considered inferior to other analytical techniques, particularly high-performance liquid chromatography, due to its lower sensitivity—primarily caused by the use of less sensitive detectors (typically UV-Vis)—reduced repeatability attributed to the small sample injection volume, and the “ageing” of the capillary’s inner surface leading to variations in electroosmotic flow, studies in the literature demonstrate that these criticisms are not entirely justified and that these issues can be resolved. In recent years, the use of modern solutions in the construction of the CE apparatus and the replacement of spectrophotometric detection with more sensitive and/or selective detectors, e.g., electrochemical, conductometric, mass spectrometry, laser-induced fluorescence, or appropriate preparation of the sample before analysis, allow for obtaining a satisfactory sensitivity and reproducibility, at the same level or sometimes better than the values obtained for the HPLC analysis.

Electromigration techniques are characterized by high efficiency and selectivity, short analysis times and low consumption of reagents. The advantage of CE is the additional possibility of analyzing substances of both low and high molecular weights, charged and neutral, with the usually uncomplicated stage of sample preparation. Moreover, the capillaries used are more durable and less susceptible to damage compared to the columns used in HPLC (introducing a sample with a complex matrix into the HPLC column carries a high risk of damaging the column), which is important, considering the costs of the analysis.

Based on the literature review, it can be concluded that, despite the fact that electromigration techniques are less popular and less frequently used, compared to HPLC, in routine laboratory analyses, they can be successfully applied to determine a wide range of substances, both ionic and neutral, included in cosmetics. The use of an appropriate technique and optimization of the process conditions allows for obtaining good sensitivity, repeatability, and resolution of the analysis, with the limits of quantification of analytes at a level sufficient to achieve values below those indicated in the regulations in force.

## Figures and Tables

**Table 1:** Methodologies for determining the cosmetic ingredients with preservation properties (L_ef_—effective length; d_i—_internal diameter).

Analyte	Matrix	Technique/ Detection	Quartz Capillary L_ef_ (cm) × d_i_ (µm)	Analysis Conditions	Ref.
MP, EP, PP, BP	Sunscreen cream and balm, hand creams, after-sun gel	CE/UV	uncoated 40 × 50	20 kV; 25 °C; 298 nm; BGE: 25 mM BB (pH = 10.0); Injection: 34.5 mbar, 5 s	[48]
MP, EP, PP, BP, SOA, BA, SA	Creams and balms	CE/UV	41 × 50	20 kV; 20 °C; 230 nm; BGE: 30 mM BB + 12% ACN; Injection: 15 s	[49]
MP, EP, PP, BP, IBP, HBZA, DHA, SOA, BA, SA	Cream based on Olivem 1000	CE/UV	40 × 50	−20 kV; 25 °C; 214 nm; BGE: 50 mM NaH_2_PO_4_ (pH = 3.0) + 100 mM SDS + 30% MeOH; Injection: 689.5 mbar, 90 s	[50]
MP, EP, PP, BP	Shampoo, hair dyes, toothpaste, shaving gel	CE/DAD	50 × 75	20 kV; 200 nm; BGE: 20 mM BB (pH 9.0) + 10% *v*/*v* MeOH); Injection: 10 s	[51]
MP, EP, PP, BP	Conditioner, balms, nail cream	CE/DAD	8.5 × 50	30 kV; 25 °C; 297 nm; BGE: 20 mM HIBA + 30 mM triethylamine + 0.3 mM HMB (pH = 10.6); Injection: 50 mbar, 30 s	[52]
MP, EP, PP	Creams, toners, gels	MEKC/UV	40 × 50	15 kV; 254 nm; BGE: 20 mM BB (pH = 9.3) + 100 mM SDS; Injection: 6 kV, 20 s	[53]
MP, EP, PP, BP	Various cosmetics	MEKC/DAD	uncoated 50 × 50	−20 kV; 25 °C; 210 nm; BGE: 50 mM H_3_PO_4_ (pH 2.28) + 100 mM SDS + 1% *v*/*v*; Injection: 50 mbar, 1 s	[12]
MP, EP, PP, BP, SA	Mask, liquids, essence, facial cleansing milk	MEKC/UV-Vis	-	15 kV; 25 °C; 200 nm; BGE: 15 mM BB (pH= 8.5) + 40 mM SDS + 0,1% *w*/*v* PEO; Injection:10 s	[54]
MP, EP, PP, BP, SOA, BA, DHA, imidourea, triclosan	Cosmetic liquids based on oil and water	MEKC/UV-Vis MEEKC/UV-Vis	MEKC: 40 × 50 MEEKC: 21 × 50	25 kV; 200 nm; MEKC: 25 °C; BGE: BB (pH 9.0) + 20 mM SDS; MEEKC: 34 °C; BGE: 89.3 wt. % BGE MEKC (pH 9.5) + 3.3 wt. % SDS + 0.8% wt. octane; Injection: 0.03 bar, 3 s	[55]
MP, EP, PP, BP, BZP	Hand cream, powder foundation	CEC/DAD	filled with 100 C18 (5 µm) 8 × 75	−30 kV; 195 nm; BGE: 5 mM ammonium formate (pH = 3.0) + 65% ACN; Injection: −12 bar, 30 s; −12 bar, 12 s	[56]

**Table 2:** Methodologies for determining cosmetic ingredients with exfoliating and moisturizing properties (L_ef—_effective length; d_i_—inner diameter).

Analyte	Matrix	Technique/ Detection	Quartz Capillary L_ef_ (cm) × d_i_ (µm)	Analysis Conditions	Ref.
Exfoliating					
Acids: formic, acetic, citric, glycolic, lactic	Cosmetic liquids, balms, and creams	CE/UV	-	−15 kV; 25 °C; 254 nm; BGE: 15 mM potassium hydrophthalate + 0.4 mM TTAB (pH = 5.6); Injection: 50 mbar, 5 s	[61]
Acids: tartaric, glycolic, lactic	Face creams, hand cream, foot cream	CE/UV	50 × 75	20 kV; 35 °C; 254 nm; BGE: 10 mM potassium hydrophthalate + 0.5 mM CTAB (pH 4.1); Injection: 34.5 mbar, 5 s	[11]
Acids: lactic, malic, tartaric, glycolic, citric, almond, salicylic	Brightening gel, exfoliating liquid and scrub	CE/DAD	40 × 50	−15 kV; 25 °C; 200 nm; BGE: 150 mM PB (pH = 7.0) + 3 mM γ-CD + 0.5 mM CTAB + 25% MeOH; Injection: 34.5 mbar, 20 s	[62]
Acids: salicylic, almond, citric, tartaric, glycolic, lactic	Cosmetic gel and liquid	CE/DAD	40 × 50	−15 kV; 25 °C; 200 nm; BGE: 200 mM PB (pH = 7.0) + 3 mM γ-CD + 0.5 mM CTAB + 25% *v*/*v* MeOH; Injection: 34.5 mbar, 20 s	[63]
Moisturizing					
Low and high molecular weight hyaluronic acid	Emulsifier Olivem 1000	MEKC/UV	uncoated 40 × 50	20 kV; 25 °C; 200 nm; BGE: 25 mM BB (pH = 9.75) + 30 mM SDS + 10% PEG; Injection: 689.5 mbar, 20 s	[64]
Hyaluronic acid	Serum	CE/DAD	uncoated 40.2 × 50	−16 kV; 25 °C; 195 nm; BGE: 200 mM PB + 200 mM butylamine + 0.5% *wt*./*v* polyethylene glycol (pH = 4.0); Injection: 34.5 mbar, 60 s	[65]
Fatty acids: palmitic, stearic, oleic, linoleic	Creams containing Brazilian nut oil	CE/DAD	uncoated 50 × 75	20 kV; 25 °C; 254 nm; BGE: 12.5 mM BB (pH = 7.0) + 12.5 mM polyoxyethylene 23-lauryl ether + 7.5 mM SDS + 35% *v*/*v* ACN; Injection: 12 mbar, 4 s	[66]
Fatty acids: palmitic, stearic, oleic, linoleic	Pumpkin seed oil cream	CE/DAD	uncoated 40 × 75	19 kV; 25 °C; 224 nm; Buffer: 15 PB (pH = 7.0) + 4 mM SDBS + 8.3 mM Brij 35 + 45% *v*/*v* ACN + 2.1% octanol; Injection: 12 mbar, 4 s	[67]
L-panthenol, dexpanthenol	Mouthwash	CE/DAD	uncoated 50 × 50	30 kV; 30 °C; BGE: 25 mM CE- β-CD in 100 mM BB (pH 9.0), Injection: 50 mbar, 10 s.	[68]

**Table 3:** Methodologies for determining the UV-absorbing ingredients of cosmetics (L_ef_—effective length; d_i_—internal diameter).

Analyte	Matrix	Technique/ Detection	Quartz Capillary L_ef_ (cm) × d_i_ (µm)	Analysis Conditions	Ref.
2,4-hydroxybenzophenone, 2,2′,4,4′-tetrahydroxybenzophenone, 2-hydroxy-4-methoxybenzophenone, 2-hydroxy-4-methoxy-5-sulfonylbenzophenone, 2,2′-dihydroxy-4,4′-dimethoxybenzophenone, 2,2′-dihydroxy-4-methoxybenzophenone, 2-hydroxy-4-octyloxybenzophenone	Sunscreen cosmetics	CE/UV	35 × 75	20 kV; 285 nm; BGE: 20 mM BB + 0.2% Tween 20; Injection: 0.5 s	[70]
4-aminobenzoic acid, 2-hydroxy-4-methoxybenzophenone-5-sulfonic acid, 2-hydroxy-4-methoxybenzophenone, 2,2′-4,4′-tetrahydroxybenzophenone, 2,2′-dihydroxy-4-methoxybenzophenone, 2,2′-dihydroxy-4,4′-dimethoxybenzophenone, 2,4-dihydroxybenzophenone, 2-hydroxy-4-methoxy-4′-methylbenzophenone	Creams	CE/UV	uncoated 52.5 × 50	25 kV; 25–26 °C; 254 nm; BGE: 20 mM BB (pH = 10.0); Injection: 8.5 nL; 5 s	[43]
Benzophenones	Oil and water-based liquid cosmetics	MEKC/UV-Vis MEEKC/UV-Vis	40 × 50	11–25 kV; 25 and 40 °C; 200 nm; BGE: MEKC: Tris (pH 9.0) + 0.3–1.5% *m*/*v* SDS MEEKC: 97.4–98.6% *v*/*v* Tris (pH 9.0) + 0.5% *m*/*v* ethyl octanoate, 1.2% m/v 1-butanol + 0.3–1.5% *m*/*v* SDS; Injection: 5 mbar, 3 s	[71]
Eusolex group UV filters	Sunscreen balm	MEEKC/DAD	52 × 50	30 kV; 25 °C; BGE: 2.2 g SDS + 0.75 g Brij 35 + 6.6 g 1-butanol + 0.8 g n-octane + 17.5 g 2-propanol + 72.1 g 10 mM BB (pH = 9.2); Injection: 50 mbar, 5 s	[72]

**Table 4:** Methodologies for determining cosmetic ingredients with antioxidant properties and dyes (L_ef_—effective length; d_i_—internal diameter).

Analyte	Matrix	Technique/ Detection	Quartz Capillary L_ef_ (cm) × d_i_(µm)	Analysis Conditions	Ref.
Antioxidants					
Propyl gallate, *t*-butyl hydroquinone	Cosmetics	CE/ED	50 × 75	20 kV; ED: 0.7 V; BGE: 5 mM BF (pH = 8.0)	[73]
Propyl gallate, *t*-butyl hydroquinone, butylated hydroxyanisole, butylated hydroxytoluene	Face mask, bath oil, sunscreen balm	MEKC/ED	75 × 25	18 kV; 95 mV (ED); BGE: 20 mM borate BGE (pH = 7.4) + 25 mM SDS; Injection: 6 s	[74]
Carnosine, nicotinamide	Whitening essence, antiglycation pill	Microfluidic CZE in COC chips/laser-induced fluorescence	-	800 V; BGE: 0.1 mM sodium tetraborate with SDS at 0.03% *w*/*w*; separation distance: 1.5 cm	[75]
L-ascorbic acid, ascorbyl 6-palmitate, magnesium ascorbyl phosphate	Cosmetics	MEKC/HPLC-UV	44.5 × 50	20 kV; 25 °C; 265 nm; BGE: 10 mM BB (pH = 9.5) + 50 mM SDS; Injection: 50 mbar, 6 s	[76]
Dyes					
Dyes: Basic Blue 99 (benzylammonium chloride), Basic Yellow 57, Basic Brown 16, Basic Brown 17, Basic Red 76	Hair coloring gels	CE/DAD	40 × 50	30 kV; 30 °C; 210 nm; BGE: 100 mM acetate buffer (acetic acid + HIBA 50:50) + 90% *v*/*v* MeOH (pH = 6.6); Injection: 50 mbar, 2 s	[77]
Dyes: Yellow Orange FCF, Acid Red 33, Tartrazine, Disodium Eosin Y Salt, Sodium Red 6 Salt, Orange II, Carmine, Rhodamine B	Lipsticks	MEKC/DAD	50 × 50	30 kV; 25 °C; 215 nm; BGE: BB (pH = 9) + 80 mM SDS; Injection: 48.6 mbar, 6 s	[78]
Aniline, p-nitroaniline, p-naphthylamine	Nail polish	Open-tubular CEC	coated with copolymer P(BMA)71- b-P(GMA)9 75 × 50	15 kV; 254 nm; BGE: 20.0 mM borax + 5.0 mM SDS (pH = 9.0); Injection: siphoned for 3 s in 15 cm height	[79]

**Table 5:** Methodologies for the determination of specific cosmetic ingredients and contaminants (L_ef_–effective length; d_i_–internal diameter).

Analyte	Matrix	Technique/Detection	Quartz Capillary L_ef_ (cm) × d_i_ (µm)	Analysis Conditions	Ref.
Hexachlorofene	Loose powder, emulsion, toner	CE/UV	40 × 75	25 kV; 25 °C; 208 nm; BGE: 20 mM BB + 10% *v*/*v* MeOH + 1 M NaOH (pH = 9.20)	[41]
Surfactants	Cosmetics and cleansing products	CE/UV	75 × 100	25 kV; 25 °C; 210 nm; BGE: 80 mM BB + 20 mM NaOH; Injection: 1 s	[44]
Ethylenediaminetetraacetic acid, ethylenediamine-disuccinic acid, iminodisuccinic acid	Shower cream, bath foam	CE/UV	50 × 50	−25 kV; 25 °C; 240 nm; BGE: 10 mM BGE MES/MOPSO + 0.25 mM TTAB (pH = 5.5); Injection: 50 mbar, 2 s	[45]
Nanosilver	Face cream	CE/DAD	-	20 kV; 410 nm; BGE: 40 mM SDS + 10 mM CAPS + 0.1% *v*/*v* MeOH (pH = 9) + 0.1% m/w mercaptosuccinic acid; Injection: 34.5 mbar, 50 s	[80]
Enantiomers of polycyclic musks: galaxolide (HHCB), traseolid (ATII), tonalide, phantolide	Body care products	CD-CE/DAD	uncoated 40 or 64 × 50	20 or 30 kV; 15 °C; 205 nm; BGE: 35 mM HP-γ-CD, 15 mM-γ-CD in 100mM CHES buffer (pH 9.0) + 5 M urea + 100 mM SDS; Injection: 50 mbar, 3 s	[81]
Copper tripeptide (GHK–Cu) complex	liposomes containing GHK–Cu complex	CE-ICP-MS/MS	70 × 75	15 kV; BGE: 5 mM Tris–HCl (pH = 7.4); Injection: 30 mbar, 5 s	[82]
Prednisone, hydrocortisone, prednisolone, hydrocortisone acetate, cortisone acetate, prednisolone acetate, triamcinolone acetonide	Body lotion; nutritive cream; mink oil cream	MEEKC/UV	50 × 50	20 kV; 25 °C; 250 nm; BGE: 3.3% SDS + 0.8% n-heptane + 6.6% n-butanol + 50 mM BB (pH 8.7); Injection: electrokinetically, 20 kV, 3 s	[83]
Lead, cadmium, mercury	Creams and lotions	CE/DAD	uncoated 40 × 50	−20 kV; 25 °C; 200 nm; BGE: 100 mM ammonium acetate (pH = 6); Injection: 34.5 mbar, 5 s	[84]
Bisphenol A, α-naphthol, β-naphthol, 2,4-dichlorophenol	Toner, balm, makeup remover, perfumes	CE/UV	65 × 50	25 kV; 20 °C; 214 nm; BGE: 30 mM BB (pH = 9.8); Injection: 25 mbar, 5 s	[85]

## Data Availability

Not applicable.

## Abbreviations

For methods:	
CE	capillary electrophoresis;
CEC	capillary electrochromatography;
CGE	capillary gel electrophoresis;
CIEF	capillary isoelectric focusing;
CITP	capillary isotachophoresis;
CZE	capillary zone electrophoresis;
DAD	diode array detector;
DLLME	dispersive liquid-liquid micro extraction;
FASI	field amplified stacking injection;
HPLC	high performance liquid chromatography;
IC	ion chromatography;
IL-DLLME	dispersive liquid–liquid microextraction;
LVSEP	large volume sample stacking using the electro-osmotic flow pump;
LVSS	large volume sample stacking;
MAE	microwave assisted extraction;
MEEKC	microemulsion electrokinetic chromatography;
MEKC	micellar electrokinetic chromatography;
SE	solvent extraction;
SFE	supercritical fluid extraction;
UV	ultraviolet detector;
Vis	visible detector.
For chemicals:	
ACN	acetonitrile;
AF	ammonium formate;
BA	benzoic acid;
BB	borate buffer;
BGE	background electrolyte;
BP	butyl paraben;
BZP	benzyl paraben;
CAPS	3-(cyclohexylamino)-1-propanesulfonic acid;
CE-β-CD	(2-carboxyethyl)- β-cyclodextrin;
CHES	2-[*N*-cyclohexylamino]ethane sulfonic acid;
CTAB	cetyltrimethylammonium bromide;
DHA	dehydroacetic acid;
EP	ethyl paraben;
HIBA	2-hydroxyisobutyric acid;
HMB	hexane-1,6-bis(trimethylammonium) bromide;
HP-γ-CD	2-hydroxylpropyl-γ-cyclodextrin;
IBP	isobutyl paraben;
MeOH	methanol;
MES	2-(*N*-morpholino)ethanesulfonic acid monohydrate;
MOPSO	3-morpholino-2-hydroxypropane-sulfonic acid;
MP	methylparaben;
PB	phosphate buffer;
PEO	poly(ethylene oxide);
PP	propyl paraben;
SA	salicylic acid;
SDBS	sodium dodecyl benzenesulfonate;
SDS	dodecyl sulfate sodium salt;
SOA	sorbic acid;
TTAB	tetradecyltrimethylammonium bromide.
